# Diagnosis-specific sickness absence among injured working-aged pedestrians: a sequence analysis

**DOI:** 10.1186/s12889-023-15259-w

**Published:** 2023-02-20

**Authors:** Linnea Kjeldgård, Helena Stigson, Eva L. Bergsten, Kristin Farrants, Emilie Friberg

**Affiliations:** 1 Division of Insurance Medicine, Department of Clinical Neuroscience, Karolinska Institutet, 171 77 Stockholm, Sweden; 2 Division of Vehicle Safety, Mechanics and Maritime Sciences, Chalmers University of Technology, Gothenburg, Sweden; 3 Folksam Research, Folksam Insurance Group, Stockholm, Sweden; 4 Faculty of Health and Occupational Studies, Department of Occupational Health Sciences and Psychology, University of Gävle, 801 76 Gävle, Sweden

**Keywords:** Sick leave, Disability pension, Pedestrians, Fall accidents, Traffic injury, Population-based

## Abstract

**Background:**

The knowledge about the long-term consequences in terms of sickness absence (SA) among pedestrians injured in a traffic-related accident, including falls, is scarce. Therefore, the aim was to explore diagnosis-specific patterns of SA during a four-year period and their association with different sociodemographic and occupational factors among all individuals of working ages who were injured as a pedestrian.

**Methods:**

A nationwide register-based study, including all individuals aged 20–59 and living in Sweden, who in 2014–2016 had in- or specialized outpatient healthcare after a new traffic-related accident as a pedestrian. Diagnosis-specific SA (> 14 days) was assessed weekly from one year before the accident up until three years after the accident. Sequence analysis was used to identify patterns (sequences) of SA, and cluster analysis to form clusters of individuals with similar sequences. Odds ratios (ORs) with 95% confidence intervals (CIs) for association of the different factors and cluster memberships were estimated by multinomial logistic regression.

**Results:**

In total, 11,432 pedestrians received healthcare due to a traffic-related accident. Eight clusters of SA patterns were identified. The largest cluster was characterized by no SA, three clusters had different SA patterns due to injury diagnoses (immediate, episodic, and later). One cluster had SA both due to injury and other diagnoses. Two clusters had SA due to other diagnoses (short-term and long-term) and one cluster mainly consisted of individuals with disability pension (DP). Compared to the cluster “No SA”, all other clusters were associated with older age, no university education, having been hospitalized, and working in health and social care. The clusters “Immediate SA”, “Episodic SA” and “Both SA due to injury and other diagnoses” were also associated with higher odds of pedestrians who sustained a fracture.

**Conclusions:**

This nationwide study of the working-aged pedestrians observed diverging patterns of SA after their accident. The largest cluster of pedestrians had no SA, and the other seven clusters had different patterns of SA in terms of diagnosis (injury and other diagnoses) and timing of SA. Differences were found between all clusters regarding sociodemographic and occupational factors. This information can contribute to the understanding of long-term consequences of road traffic accidents.

**Supplementary Information:**

The online version contains supplementary material available at 10.1186/s12889-023-15259-w.

## Background

Walking as physical activity and active transportation is good for public health and accordingly encouraged by different stakeholders [1–3]. However, walking in a road traffic environment (i.e. being a pedestrian) also involves some risk. Pedestrians can slip, trip, stumble, or collide with other road users. Globally, about a fifth of all fatalities within the road transport system are represented by pedestrians [4, 5]. The UN’s global sustainable development goals strive both for more sustainable and safer transportation [5, 6]. Likewise, the Swedish Vision Zero has the long-term vision that no one should die or suffer injuries leading to long-term consequences within the road transport system [7].

Being injured in a road traffic accident may affect the individual’s work ability and lead to sickness absence (SA) or disability pension (DP). We have previously shown that many (20%) injured pedestrians had a new SA spell in connection to the accident [8]. However, the knowledge of long-term SA and the patterns of SA among injured pedestrians is limited. In addition, diverse work requirements for different types of work (e.g. occupational sectors and type of occupation) could impact the injured pedestrian’s need of SA or DP. However, the impact of the occupational factors in relation to SA and DP among pedestrians involved in a traffic-related accident have, to the best of our knowledge, not yet been explored.

To get a broader understanding of the long-term consequences in terms of SA among pedestrians injured in traffic-related accidents, including falls within the road transport system, the aim of this study was to explore patterns of diagnosis-specific (i.e. injury diagnoses or other diagnoses) SA during a four-years period and their association with different background factors including occupational factors among all individuals of working ages who were injured as a pedestrian.

## Methods

A prospective cohort study was conducted, including all pedestrians, 20–59 years of age (which is to say within working ages throughout the follow-up) who had at least one hospitalization or visit to specialized outpatient healthcare due to an injury (International Statistical Classification of Diseases and Related Health Problems; ICD-10 [9]: S00-T88) sustained in a new traffic-related accident (including falls and bumping into other pedestrians) (ICD-10: V01-V09, W00.4, W01.4, W02.4, W03.4, W04.4, W05.4, W10.4, W15.4, W17.4, W18.4, W19.4, W51.4) between 1 January 2014 and 31 December 2016 (*n* = 12,870) and living in Sweden. Individuals who were living in Sweden the 31 December the year before the accident and did not have any traffic related in- or out-patient healthcare (ICD-10: V01-V99, W00.4, W01.4, W02.4, W03.4, W04.4, W05.4, W10.4, W15.4, W17.4, W18.4, W19.4, W51.4) during two years before the accident, were included. The date of the accident, denoted as T_0,_ refers to the first date of the in- or specialized outpatient healthcare visit/hospitalization, as the actual date of their accident/fall is not included in the registers.

The SA-status of the study population was assessed weekly during a period of four years; one year before and three years after the accident date, T_0_. Individuals who died (*n* = 213) or emigrated (*n* = 58) during the follow-up period were excluded. In addition, those with disability pension (DP) (full- or part-time) during all of the 209 weeks of the four-year study period were excluded (*n* = 1167). This resulted in a study population of 11,432 injured pedestrians.

Microdata from several nationwide registers were used and linked at the individual level, using the unique personal identity number assigned to all residents in Sweden [10].

- The in- and specialized outpatient registers, from the *National Board of Health and Welfare*, were used to identify the study population as well as for medical information related to the injury.

- The Cause of Death Register, from the *National Board of Health and Welfare*, was used to identify those who had died within three years from the accident date.

- The Longitudinal Integration Database for Health Insurance and Labour Market Studies (LISA), from *Statistics Sweden*, was used to identify the source population, all individuals living in Sweden 31 December the year before the accident, and information for the study population regarding sociodemographic factors (sex, age, educational level, country of birth, type of living area, and marital status) and occupational factors (occupational sector, private/public, and type of occupation) also measured 31 December the year before the accident.

- Micro-data for Analyses of the Social Insurance (MiDAS), from the *Swedish Social Insurance Agency*, was used for information on the dates and diagnoses of SA and DP.

Reference groups for the below factors were chosen based on size of the groups and expected proportions with SA, with larger groups or groups expected to have lower proportions of SA being used as reference groups.

The type of accident was categorized into the following six groups: collision with pedestrian/bicyclist (V01, W03.4, W04.4, W51.4); collision with motor vehicle (V02-V06, V09.0, V09.2); unspecified (V09.1, V09.3, V09.9, W19.4); fall: snow and ice, street and highway (W00.4); fall: slipping, tripping, and stumbling, street and highway (W01.4) (*reference group*); and fall: other, street and highway (W02.4, W05.4, W10.4, W15.4, W17.4, W18.4).

The main diagnosis and the secondary diagnoses for the healthcare visit/hospitalization were categorized using a modified version of the Barell matrix [11], into categories of injured body region and type of injury. Most of the pedestrian had only one injury diagnosis recorded, but for those individuals who had several, the main diagnosis was prioritized before any of the secondary diagnoses. Some pedestrians had up to three visits/hospitalizations at T_0._ In these cases, the injuries requiring inpatient healthcare were prioritized over the injuries treated in outpatient healthcare. In addition, an injury with ICD10: S00-S99 was prioritized over an injury with ICD10: T00-T88.

The injured body region was categorized into the following six groups: head, face, and neck (*reference group*); vertebral column and spinal cord; torso; upper extremities; lower extremities; and “other and unspecified”. The type of injury was categorized into six groups: fracture; dislocation; sprains and strains; internal (brain, spinal cord, and other internal organs); external (open wounds, contusions, and superficial injuries) (*reference group*); and “other and unspecified”. Similar categorizations have been used in recent studies on injuries among different groups of road traffic users [8, 12–15].

Healthcare at T_0_ was also used to categorize inpatient healthcare: no (only specialized outpatient healthcare) (*reference group*); yes. If someone had both specialized outpatient healthcare and inpatient healthcare they were categorized as inpatient healthcare.

The sociodemographic factors were categorized as: sex (women; men (*reference group*)), age group (20–24; 25–34 (*reference group*); 35–44; 45–54; 55–59 years), level of education (elementary school (≤ 9 years including missing); high school (9–12 years); university/college (> 12 years) (*reference group*)), country of birth (Sweden (*reference group*); not Sweden), type of living area (cities (*reference group*); town and suburbs; rural areas), marital status (married (*reference group*); not married). These have all been demonstrated to be common risk factors for SA in general [16] and after a traffic related injury [8, 12–15].

Several occupational factors were also included. Occupational sector was categorized according to the Swedish Standard of Industrial Classification SNI 2007 (Manufacturing, agriculture, forestry & fishing (G01, G02, G03); Construction (G04); Trade, transport, hotels & restaurants (G05, G06, G07); Finance, communication & cultural service (G08, G09, G10, G11, G12, G15) (*reference group*); Education (G13); Health & social care (G14); Not in work/Unknown (G99)), Private/public employer (private (*reference group*); public; Not in work/Unknown), and also type of occupation, according to the Swedish Standard for Occupational Classification SSYK (white collar (*reference group*); blue collar; Not in work/Unknown).

Lastly, season was based on T_0_ and categorized as winter (December, January, February), spring (Mars, April, May), summer (June, July, August) (*reference group*), autumn (September, October, November).

All individuals living in Sweden, ≥ 16 years old, and with income from work, unemployment, or parental-leave benefits can apply for SA benefits from the Social Insurance Agency if having a disease or injury that leads to reduced work capacity [17]. The first day of a SA spell is an unreimbursed qualifying day (more days for self-employed). A physician’s certificate is required after day 7. For employees, day 2–14 are reimbursed by the employer [17]. For others, e.g., unemployed, the Social Insurance Agency administers the benefits from the second day of SA, with information on shorter SA spells available for these individuals. Therefore, in order not to introduce a bias, only information on SA spells > 14 days was used. All individuals aged 19–64 can be granted DP if disease or injury leads to long-term or permanent work incapacity. Both SA and DP can be granted for full- or part-time (100, 75, 50, 25%) of ordinary work hours. Accordingly, someone on part-time DP can at the same time have part-time SA. For young individuals (19–30 years old) DP can be time limited. In general, it is very uncommon but possible to go from DP to non-DP.

Weekly states of SA were defined for the study population. To do this, the four years of follow-up were divided into 209 weeks, 52 weeks prior (W_−52_) through 156 weeks after (W_+156_) the week of the accident W_0_, defined as T_0_ ± 3 days. For each week, individuals were assigned a state (one out of four non overlapping states) based on their SA situation during that week: No SA or DP (no SA or DP during the week); SA due to injury diagnosis (any SA due to an injury diagnosis (ICD10: S00-T98) during the week, and no DP); SA due to other diagnoses (any SA due to other diagnoses than injuries, no DP, and no SA due to an injury diagnosis during the week); and DP (any DP, regardless of extent or diagnosis, during the week).

### Statistical analyses

Descriptive statistics of the study population were conducted including stratified by sex.

The patterns of SA states during a four-year period (from 1 year before and through 3 years after W_0_ (W_−52_ to W_+156_)) were analyzed using sequence analysis with TraMineR in R [18]. Thereafter, cluster analysis with optimal matching spell algorithm[19] was used to identify different clusters of individuals who had similar sequences of SA-states. A cluster tree and several measures of cluster partition quality [18] were used to choose the number of clusters (Table A.1). Density plots and index plots for each cluster are shown.

**Table 1:** Characteristics of all pedestrians aged 20–59 with a road traffic injury (including falls) in 2014–2016, by sex

	All		Women		Men	
	n	%^1^	n	%^1^	n	%^1^
**All**	11,432		6212	54.34^2^	5220	45.66^2^
**Age group, years**						
20–24	1737	15.19	752	12.11	985	18.87
25–34	2306	20.17	1093	17.59	1213	23.24
35–44	2243	19.62	1156	18.61	1087	20.82
45–54	3144	27.50	1867	30.05	1277	24.46
55–59	2002	17.51	1344	21.64	658	12.61
**Level of education**						
Elementary school	1778	15.55	770	12.40	1008	19.31
High school	5796	50.70	2925	47.09	2871	55.00
University/College	3858	33.75	2517	40.52	1341	25.69
**Country of birth**						
Sweden	9200	80.48	4983	80.22	4217	80.79
Not Sweden	2232	19.52	1229	19.78	1003	19.21
**Type of living area**						
Cities	4752	41.57	2585	41.61	2167	41.51
Towns and suburbs	4723	41.31	2597	41.81	2126	40.73
Rural areas	1957	17.12	1030	16.58	927	17.76
**Married**						
No	7466	65.31	3755	60.45	3711	71.09
Yes	3966	34.69	2457	39.55	1509	28.91
**Type of accident**						
Collision with pedestrian/bicyclist	591	5.17	286	4.60	305	5.84
Collision with motor vehicle	1595	13.95	734	11.82	861	16.49
Unspecified	1166	10.20	547	8.81	619	11.86
Fall: snow and ice	2529	22.12	1582	25.47	947	18.14
Fall: slipping, tripping, and stumbling	4136	36.18	2491	40.10	1645	31.51
Fall: other	1415	12.38	572	9.21	843	16.15
**Inpatient healthcare**						
No	9923	86.80	5442	87.60	4481	85.84
Yes	1509	13.20	770	12.40	739	14.16
**Type of injury**						
Fracture	4587	40.12	2648	42.63	1939	37.15
Dislocation	366	3.20	145	2.33	221	4.23
Sprains and strains	1724	15.08	932	15.00	792	15.17
Internal	794	6.95	404	6.50	390	7.47
External	3749	32.79	1961	31.57	1788	34.25
Other and unspecified	212	1.85	122	1.96	90	1.72
**Injured body region**						
Head, face and neck	2345	20.51	1146	18.45	1199	22.97
Vertebral column and spinal cord	215	1.88	107	1.72	108	2.07
Torso	630	5.51	295	4.75	335	6.42
Upper extremities	4005	35.03	2308	37.15	1697	32.51
Lower extremities	4185	36.61	2330	37.51	1855	35.54
Other and unspecified	52	0.45	26	0.42	26	0.50
**Season**						
Winter	4133	36.15	2444	39.34	1689	32.36
Spring	2405	21.04	1231	19.82	1174	22.49
Summer	2329	20.37	1135	18.27	1194	22.87
Autumn	2565	22.44	1402	22.57	1163	22.28
**Year of accident**						
2014	3393	29.68	1788	28.78	1605	30.75
2015	3956	34.60	2172	34.96	1784	34.18
2016	4083	35.72	2252	36.25	1831	35.08
**Occupational sector**						
Manufacturing, agriculture, forestry & fishing	1143	10.00	309	4.97	834	15.98
Construction	586	5.13	63	1.01	523	10.02
Trade, transport, hotels & restaurants	2160	18.89	886	14.26	1274	24.41
Finance, communication & cultural service	2897	25.34	1568	25.24	1329	25.46
Education	1035	9.05	836	13.46	199	3.81
Health & social care	2076	18.16	1792	28.85	284	5.44
Not in work/Unknown	1535	13.43	758	12.20	777	14.89
**Private/Public**						
Private sector	6118	53.52	2651	42.68	3467	66.42
Public sector	2946	25.77	2390	38.47	556	10.65
Not in work/Unknown	2368	20.71	1171	18.85	1197	22.93
**Type of occupation**						
White collar	4539	39.70	3219	51.82	1320	25.29
Blue collar	3295	28.82	1295	20.85	2000	38.31
Not in work/Unknown	3598	31.47	1698	27.33	1900	36.40

^1^ column percent

^2^ row percent

Multinomial logistic regression models were used to analyze the association between sociodemographic and occupational factors, type of accident, type of healthcare, type of injury, injured body region, and SA-clusters. Crude and adjusted odds ratios (OR) and 95% confidence intervals (CI) were calculated. Sensitivity analyses were conducted including the 1167 individuals who had DP during the entire follow-up. The statistical analyses were performed using SAS (version 9.4) and R (version 3.6.1).

## Results

There were 11,432 pedestrians with in- or specialized out-patient healthcare due to a new traffic accident including fall accidents 2014–2016 aged 20–59 years (Table 1). The median age was 45 years among women and 39 years among men. The most common type of accident was fall: slipping, tripping, and stumbling, followed by fall: snow and ice. The most common types of injuries were, fractures, external injuries, and sprains and strains injuries. A quarter of the injured pedestrians worked in Finance, communication & cultural service. Among women, 29% worked in Health & social care and 14% worked in Trade, transport, hotels & restaurants; the corresponding numbers in men were 5%, and 24% respectively.

**Fig. 1:**
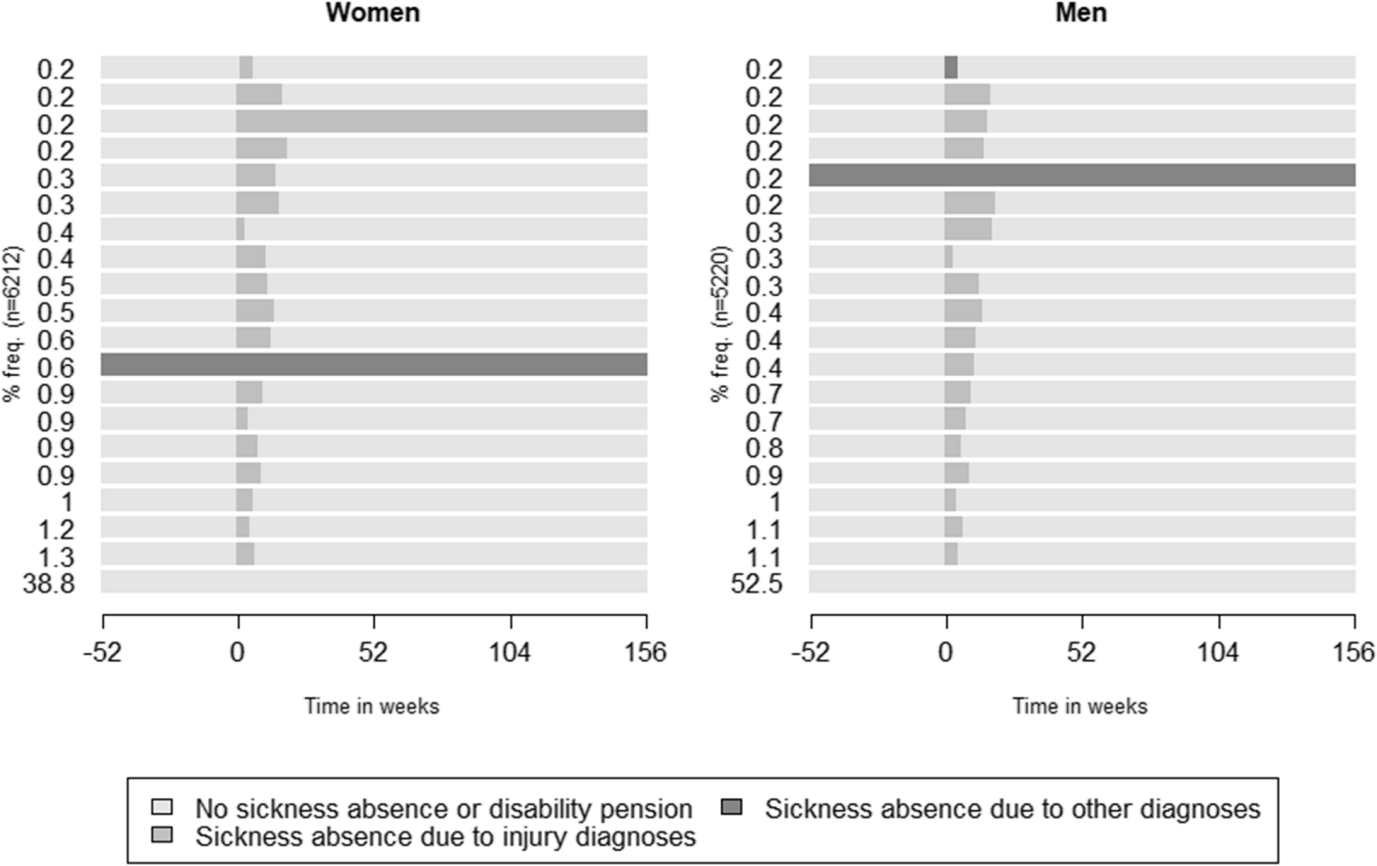
The 20 most common sequences of sickness absence states/week from one year before until three years after (W_-52_ to W_+156_) the week of the pedestrian accident (W_0_) (marked as week 0 in the figure) for women and men

Diverse patterns of SA during the study period were observed among the pedestrians (4358 unique sequences). By far the most common sequence observed, for both women and men, was to have no SA or DP during all four years (W_−52_ to W_+156_) (38.8% for women and 52.5% for men) (Fig. 1). Several of the most common sequences included no SA prior to the accident and then SA of various durations due to injury diagnoses starting from the week of the accident (W_0_). Another common sequence observed among the individuals was to have SA due to other diagnosis (i.e. not injury) during the entire study period (0.6% among women and 0.2% among men) (Fig. 1).

**Fig. 2:**
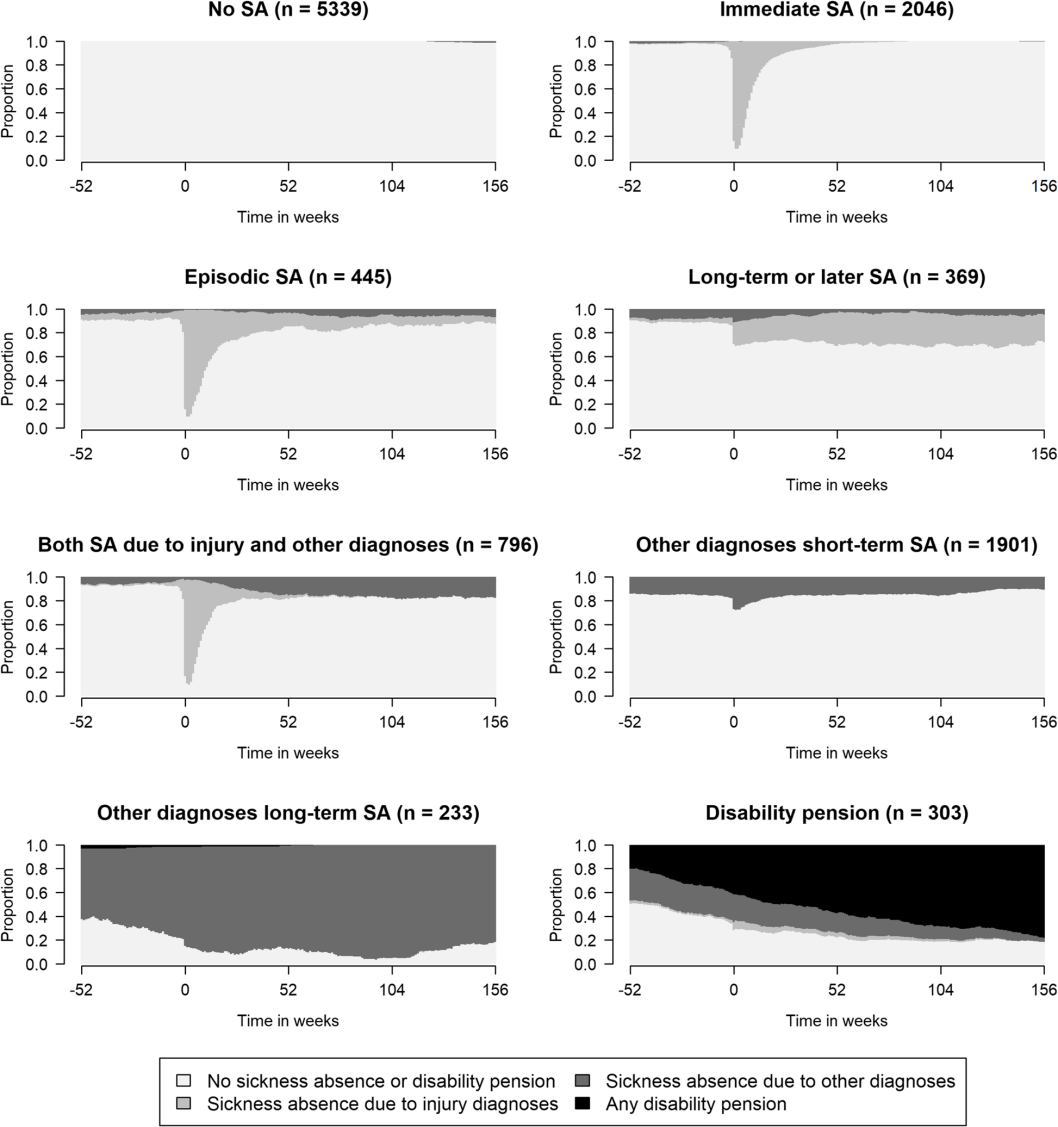
Density plots of sickness absence (SA) states/week during the year before through three years after (W_−52_ to W_+156_) the week of the pedestrian accident (marked with 0 in the figure), for the eight identified clusters. The number of individuals in each cluster are stated in each cluster heading

**Fig. 3:**
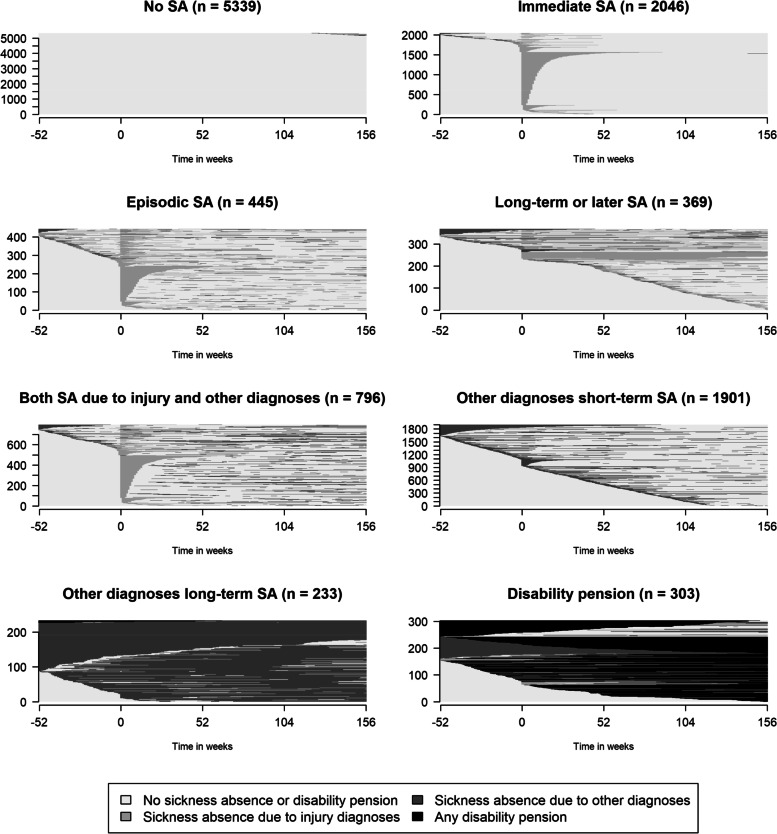
Index plots of sickness absence (SA) states/week during the year before through three years after (W_−52_ to W_+156_) the week of the pedestrian accident (marked with 0 in the figure), for the eight identified clusters. The number of individuals in each cluster are stated in each cluster heading and every line represents one individual. The plots are ordered by the first state in the sequences

Cluster analysis was then used to form groups of individuals who had similar sequences. The cluster partition quality measures for the different numbers of clusters are presented in the appendix (Table A.1). Eight clusters were identified: “No SA” (including 47% of the study population), “Immediate SA” (18%), “Episodic SA” (4%), “Long-term or later SA” (3%), “Both SA due to injury and other diagnoses” (7%), “Other diagnoses short-term SA” (17%), “Other diagnoses long-term SA” (2%), and “Disability pension” (3%). In the description of the clusters, SA is referred to SA due to an injury diagnosis unless otherwise stated. The eight clusters are illustrated using density plots in Fig. 2 and using index plots in Fig. 3.

Characteristics of each of the eight identified clusters are summarised in Table 2 in terms of sequences, sociodemographic characteristics, occupational characteristics and injury related characteristics.

**Table 2:** Summary of sociodemographic, injury, and occupational characteristics in the eight identified clusters of SA sequences

Cluster	1. No SA	2. Immediate SA	3. Episodic SA	4. Long-term or later SA	5. Both SA due to injury and other diagnoses	6. Other diagnoses short-term SA	7. Other diagnoses long-term SA	8. Disability pension
N	5339 individuals	2046 individuals	445 individuals	369 individuals	796 individuals	1901 individuals	233 individuals	303 individuals
Characterisation of SA sequences	No SA or DP during the entire study period	SA due to injury diagnoses in connection to the accident	Two or more SA spells due to injury diagnoses, one at the time of the accident and one prior to or later during the study period	One or several SA spells due to injury diagnoses later during follow-up	One SA spell due to an injury diagnosis starting in connection to the accident and also one or several SA spells due to other diagnoses during the study period	One or several short-term SA spells due to other diagnoses spread out during the study period	Long-term SA due to other diagnoses	DP
Characterisation of sociodemographic differences	More young men More individuals not born in Sweden, living in cities, and with higher levels of education	More women. More individuals born in Sweden, married, and living in towns or rural areas	More older women More often married, and living in towns or rural areas	More older men More often married, born in Sweden, and with lower levels of education	More older women More often married, born in Sweden, and living in towns or rural areas	More women More often born in Sweden	More older women More often born in Sweden, living in cities, and with lower levels of education	More often either younger and older individuals More often unmarried, living in towns or rural areas, and with lower levels of education
Characterisation of occupation differences	More often not in work/unknown work	More often working in Construction, Trade, transport, hotels & restaurants, and Health & social care	More often working in Construction, and Health & social care. More often blue-collar worker	More often working in Health & social care. More often blue-collar worker	More often working in Education, and Health & social care. More often white-collar worker	More often working in Education, and Health & social care	More often working in Health & social care. More often not in work/unknown work	More often working in Health & social care. More often not in work/unknown work
Characterisation of the injuries	More often external injuries and injuries to head, face and neck	More often fractures and injuries to upper and lower extremities	More often fractures and injuries to upper and lower extremities	More often external injuries and injuries to vertebral column & spinal cord	More often fractures and injuries to upper and lower extremities	More often injuries to torso and vertebral column & spinal cord		More often internal injuries and injuries to head, face and neck

Almost all of the 5339 individuals in the cluster “No SA” were not on SA or DP during the entire follow up. In this cluster there were 53% men and 8% were identified through the in-patient healthcare. The most common type of injuries among individuals belonging to this cluster were external injuries (41%) and fractures (29%). Characteristics of the clusters and adjusted ORs for the other clusters compared to the cluster “No SA” are presented in Table 3. Descriptive statistics of all factors of the clusters and unadjusted and adjusted ORs can be found in the appendix (Table A.2 and Table A.3).

**Table 3:** Distributions and adjusted odds ratios (ORs) and 95% confidence intervals (CIs) for different factors in each of the eight clusters of sickness absence (SA) sequences spanning 1 year before to 3 years after the pedestrian accident (W_−52_ to W_+156_) among 11,432 individuals aged 20–59 years injured in a traffic-related accident in 2014–2016, using the cluster “No SA” as the reference

	1. No SA	2. Immediate SA		3. Episodic SA			4. Long-term or later SA			5. Both SA due to injury and other diagnoses			6. Other diagnoses short-term SA			7. Other diagnoses long-term SA			8. Disability pension		
		n (%)	Adj OR^1^ (95% CI)	n (%)	Adj OR^1^ (95% CI)		n (%)	Adj OR^1^ (95% CI)		n (%)	Adj OR^1^ (95% CI)		n (%)	Adj OR^1^ (95% CI)		n (%)	Adj OR^1^ (95% CI)		n (%)	Adj OR^1^ (95% CI)	
**All**	5339 (46.7)	2046 (17.9)		445 (3.89)			369 (3.23)			796 (6.96)			1901 (16.63)			233 (2.04)			303 (2.65)		
**Type of accident**																					
Collision with pedestrian/bicyclist	307 (5.75)	89 (4.35)	1.28 (0.96–1.70)	21 (4.72)	1.36 (0.83–2.24)		28 (7.59)	1.60 (1.03–2.48)		44 (5.53)	1.70 (1.18–2.45)		79 (4.16)	0.78 (0.60–1.03)		10 (4.29)	0.86 (0.44–1.69)		13 (4.29)	0.87 (0.47–1.60)	
Collision with motor vehicle	777 (14.55)	286 (13.98)	1.54 (1.26–1.87)	57 (12.81)	1.39 (0.98–1.98)		59 (15.99)	1.23 (0.87–1.73)		79 (9.92)	1.12 (0.84–1.50)		256 (13.47)	0.95 (0.79–1.14)		33 (14.16)	0.87 (0.56–1.35)		48 (15.84)	0.91 (0.62–1.33)	
Unspecified	587 (10.99)	163 (7.97)	1.06 (0.85–1.32)	42 (9.44)	1.23 (0.85–1.79)		35 (9.49)	0.93 (0.63–1.39)		61 (7.66)	1.03 (0.76–1.41)		220 (11.57)	1.08 (0.90–1.30)		25 (10.73)	0.93 (0.59–1.49)		33 (10.89)	0.93 (0.61–1.42)	
Fall: snow and ice	1005 (18.82)	600 (29.33)	1.25 (1.05–1.48)	122 (27.42)	1.20 (0.89–1.61)		78 (21.14)	1.08 (0.77–1.50)		249 (31.28)	1.25 (1.00–1.57)		370 (19.46)	0.93 (0.79–1.11)		47 (20.17)	0.79 (0.52–1.19)		58 (19.14)	0.85 (0.58–1.24)	
Fall: slipping, tripping, and stumbling	1938 (36.30)	670 (32.75)	ref	150 (33.71)	ref		130 (35.23)	ref		279 (35.05)	ref		753 (39.61)	ref		102 (43.78)	ref		114 (37.62)	ref	
Fall: other	725 (13.58)	238 (11.63)	1.06 (0.87–1.29)	53 (11.91)	1.12 (0.79–1.58)		39 (10.57)	0.86 (0.59–1.25)		84 (10.55)	1.10 (0.83–1.45)		223 (11.73)	0.97 (0.81–1.16)		16 (6.87)	0.57 (0.33–0.99)		37 (12.21)	0.93 (0.62–1.40)	
**Type of injury**																					
Fracture	1549 (29.01)	1477 (72.19)	6.81 (5.71–8.13)	290 (65.17)	4.91 (3.57–6.75)		109 (29.54)	0.91 (0.68–1.23)		503 (63.19)	4.65 (3.63–5.96)		464 (24.41)	0.73 (0.63–0.85)		86 (36.91)	0.94 (0.66–1.35)		109 (35.97)	1.11 (0.80–1.54)	
Dislocation	177 (3.32)	73 (3.57)	2.98 (2.13–4.15)	16 (3.60)	2.37 (1.29–4.35)		13 (3.52)	0.94 (0.51–1.74)		29 (3.64)	2.60 (1.63–4.16)		48 (2.52)	0.75 (0.53–1.06)		-^2^	-^2^		-^2^	-^2^	
Sprains and strains	890 (16.67)	182 (8.90)	1.45 (1.15–1.83)	57 (12.81)	1.69 (1.13–2.52)		70 (18.97)	1.12 (0.79–1.58)		119 (14.95)	1.90 (1.41–2.57)		353 (18.57)	0.93 (0.78–1.10)		23 (9.87)	0.62 (0.37–1.03)		30 (9.90)	0.90 (0.56–1.43)	
Internal	392 (7.34)	82 (4.01)	3.08 (2.17–4.37)	17 (3.82)	3.04 (1.52–6.08)		33 (8.94)	1.32 (0.82–2.11)		34 (4.27)	3.10 (1.86–5.17)		172 (9.05)	1.27 (1.01–1.61)		20 (8.58)	1.57 (0.86–2.87)		44 (14.52)	1.93 (1.23–3.03)	
External	2213 (41.45)	220 (10.75)	ref	58 (13.03)	ref		136 (36.86)	ref		103 (12.94)	ref		820 (43.14)	ref		88 (37.77)	ref		ref	ref	
Other and unspecified	118 (2.21)	12 (0.59)	1.44 (0.76–2.75)	-^2^	-^2^		-^2^	-^2^		-^2^	-^2^		44 (2.31)	1.15 (0.79–1.67)		9 (3.86)	2.38 (1.09–5.18)		-^2^	-^2^	
**Injured body region**																					
Head, face and neck	1394 (26.11)	155 (7.58)	ref	32 (7.19)	ref		83 (22.49)	ref		61 (7.66)	ref		474 (24.93)	ref		49 (21.03)	ref		97 (32.01)	ref	
Vertebral column and spinal cord	89 (1.67)	35 (1.71)	1.53 (0.92–2.55)	10 (2.25)	2.74 (1.16–6.46)		15 (4.07)	3.13 (1.59–6.15)		13 (1.63)	1.79 (0.86–3.70)		41 (2.16)	1.77 (1.16–2.72)		-^2^	-^2^		-^2^	-^2^	
Torso	312 (5.84)	68 (3.32)	1.41 (0.97–2.04)	18 (4.04)	2.11 (1.08–4.12)		22 (5.96)	1.27 (0.75–2.13)		31 (3.89)	1.98 (1.18–3.33)		147 (7.73)	1.64 (1.28–2.09)		15 (6.44)	1.65 (0.87–3.15)		17 (5.61)	0.90 (0.50–1.62)	
Upper extremities	1654 (30.98)	938 (45.85)	3.26 (2.48–4.28)	206 (46.29)	4.44 (2.60–7.58)		129 (34.96)	1.74 (1.20–2.52)		344 (43.22)	3.27 (2.18–4.92)		557 (29.30)	1.24 (1.03–1.49)		84 (36.05)	2.01 (1.25–3.26)		93 (30.69)	1.21 (0.81–1.81)	
Lower extremities	1856 (34.76)	847 (41.40)	3.26 (2.49–4.28)	179 (40.22)	4.10 (2.41–6.99)		119 (32.25)	1.32 (0.90–1.92)		347 (43.59)	3.65 (2.44–5.48)		674 (35.46)	1.26 (1.05–1.52)		76 (32.62)	1.64 (1.01–2.65)		87 (28.71)	0.93 (0.62–1.41)	
Other and unspecified	34 (0.64)	-^2^	-^2^	-^2^	-^2^		-^2^	-^2^		-^2^	-^2^		-^2^	-^2^		-^2^	-^2^		-^2^	-^2^	
**Occupational sector**																					
Manufacturing, agriculture, forestry & fishing	556 (10.41)	230 (11.24)	0.97 (0.78–1.20)	48 (10.79)	1.11 (0.74–1.65)		49 (13.28)	1.10 (0.74–1.63)		77 (9.67)	0.96 (0.70–1.31)		165 (8.68)	0.85 (0.68–1.06)		-^2^	-^2^		12 (3.96)	0.52 (0.27–1.01)	
Construction	240 (4.50)	147 (7.18)	1.62 (1.24–2.13)	40 (8.99)	2.35 (1.51–3.65)		29 (7.86)	1.46 (0.91–2.35)		38 (4.77)	1.38 (0.92–2.09)		79 (4.16)	1.04 (0.77–1.39)		-^2^	-^2^		-^2^	-^2^	
Trade, transport, hotels & restaurants	993 (18.60)	427 (20.87)	1.20 (1.00–1.44)	85 (19.10)	1.25 (0.89–1.75)		63 (17.07)	0.86 (0.60–1.24)		150 (18.84)	1.22 (0.94–1.59)		386 (20.31)	1.12 (0.94–1.32)		34 (14.59)	1.17 (0.72–1.91)		22 (7.26)	0.51 (0.30–0.87)	
Finance, communication & cultural service	1447 (27.10)	520 (25.42)	ref	95 (21.35)	ref		85 (23.04)	ref		178 (22.36)	ref		475 (24.99)	ref		45 (19.31)	ref		52 (17.16)	ref	
Education	409 (7.66)	210 (10.26)	1.19 (0.94–1.51)	44 (9.89)	1.20 (0.79–1.83)		33 (8.94)	1.41 (0.89–2.25)		96 (12.06)	1.48 (1.08–2.02)		215 (11.31)	1.45 (1.16–1.80)		21 (9.01)	1.45 (0.80–2.61)		-^2^	-^2^	
Health & social care	656 (12.29)	456 (22.29)	1.85 (1.53–2.25)	124 (27.87)	2.45 (1.76–3.41)		75 (20.33)	1.97 (1.36–2.86)		234 (29.40)	2.38 (1.85–3.07)		443 (23.30)	1.73 (1.44–2.07)		46 (19.74)	1.99 (1.24–3.22)		42 (13.86)	1.75 (1.10–2.79)	
Not in work/Unknown	1038 (19.44)	56 (2.74)	0.45 (0.31–0.65)	9 (2.02)	0.32 (0.14–0.72)		35 (9.49)	0.99 (0.57–1.73)		23 (2.89)	0.48 (0.27–0.84)		138 (7.26)	0.61 (0.47–0.81)		75 (32.19)	2.94 (1.57–5.52)		161 (53.14)	1.63 (1.06–2.52)	
**Type of occupation**																					
White collar	1974 (36.97)	919 (44.92)	ref	181 (40.67)	ref		133 (36.04)	ref		377 (47.36)	ref		828 (43.56)	ref		72 (30.90)	ref		55 (18.15)	ref	
Blue collar	1314 (24.61)	733 (35.83)	1.93 (1.63–2.27)	175 (39.33)	2.51 (1.88–3.34)		141 (38.21)	1.86 (1.37–2.52)		277 (34.80)	2.03 (1.62–2.53)		555 (29.20)	1.35 (1.15–1.58)		51 (21.89)	1.99 (1.29–3.07)		49 (16.17)	1.48 (0.94–2.33)	
Not in work/Unknown	2051 (38.42)	394 (19.26)	1.05 (0.87–1.26)	89 (20.00)	1.42 (1.02–1.96)		95 (25.75)	1.17 (0.83–1.66)		142 (17.84)	1.05 (0.81–1.35)		518 (27.25)	1.23 (1.04–1.46)		110 (47.21)	2.48 (1.62–3.78)		199 (65.68)	2.13 (1.38–3.29)	

^1^ Adjusted for: Sex, Age group, Level of education, Country of birth, Type of living area, Married, Type of accident, Inpatient healthcare, Type of injury, Injured body region, Season, Year of accident, Occupational sector, Private/Public, and Type of occupation

^2^ Too few: ≤ 8 individuals

All clusters other than “No SA” were associated with older age, no university education, having been hospitalized, and working in health and social care.

The cluster “Immediate SA” was associated with the types of accidents collision with motor vehicle and Fall: slipping tripping and stumbling (OR (95% CI): 1.54 (1.26–1.87), and 1.25 (1.05–1.48) respectively). Whereas the clusters “Long-term or later SA” and “Both SA due to injury and other diagnoses” were associated with injuries sustained in a collision with pedestrian/bicyclists (1.60 (1.03—2.48), and 1.70 (1.18—2.45) respectively).

Fractures, dislocations, sprains and strains, and internal injuries had high OR for the clusters “Immediate SA”, “Episodic SA”, and “Both SA due to injury and other diagnoses”.

Upper extremities had high OR for all cluster but “Disability pension”. Lower extremities had high OR for all cluster but “Disability pension” and “Long-term or later SA”. Injuries to the torso were associated with the clusters “Episodic SA”, “Both SA due to injury and other diagnoses”, and “Other diagnoses short-term SA”. Injuries to the vertebral column and spinal cord were associated with the clusters “Episodic SA”, “Long-term or later SA”, and “Other diagnoses short-term SA”.

Regarding occupational factors, blue-collar work was associated with all clusters but “Disability pension” compared to the cluster “No SA” and working in Health & social care was associated with all clusters compared to “No SA”. Working in Construction had high ORs for being in the clusters “Immediate SA” (1.62 (1.24–2.13)) and “Episodic SA” (2.35 (1.51–3.65)), whereas working in Education had high ORs for being in the clusters “Both SA due to injury and other diagnoses” (1.48 (1.08–2.02)) and “Other diagnoses short-term SA” (1.45 (1.16–1.80)).

Sensitivity analyses including the 1167 individuals who had DP during the entire follow-up did not alter the results substantially (data not shown).

## Discussion

In this nationwide register study exploring diagnosis-specific patterns of SA among injured pedestrians there were in total 11,432 working-aged pedestrians that received in- or specialized outpatient healthcare due to a traffic-related accident. Of them, 71% were due to falls and a third of the falls related to snow and ice. Eight different clusters of SA were identified with different patterns of SA due to an injury diagnosis or whether due to other diagnoses. Compared to the cluster “No SA” all the other clusters were associated with older age, no university education, having been hospitalized, and working in health and social care. The clusters “Immediate SA”, “Episodic SA” and “Both SA due to injury and other diagnoses” were also associated with e.g. higher odds of individuals who sustained a fracture.

The most common injuries were fractures and external injuries, which has also been reported for pedestrians in previous studies [1, 8, 20, 21]. Injuries to the lower and upper extremities and to the head, face and neck were the most common type of injured body region, also in line with previous studies [1, 8, 20, 21]. In contrast, the proportion of injuries from falls due to snow and ice was lower in the present study compared to a previous study conducted during 2010, 22% compared to 36% of all pedestrian accidents, respectively [8]. This could partly be explained by that the winter seasons during 2010 were colder and with more snowfall than during the rather mild winters when the present study was conducted in 2014–2016 [22–24]. This difference in proportion of accidents related to snow and ice highlights the importance of road environment maintenance during the winter season to reduce the risk for these types of accidents, which is also called for by authors of other studies [25, 26].

The present study showed a rather diverse population is involved in pedestrian road traffic accidents. Higher proportions of older women yet more younger men were injured pedestrians. Women were more often injured in falls due to slipping, tripping, and stumbling as well as falls due to snow and ice, while men were more often injured in falls due to other reasons and collisions with a motor vehicle. In addition, more women were working in Health & social care and more men were working in Trade, transport, hotels & restaurants. Moreover, a higher proportion of women had university/college education than men. These differences among the injured pedestrians are important to consider, as suggest different measures might be called for in order to reduce the number of individuals involved in a pedestrian accident for women and men. For example, personal devices such as reflectors, to prevent collisions with other road traffic groups, and anti-slip shoes to prevent falls due to snow and ice, or related to the traffic environment such as gritting/salting slippery roads or improving the separation between road user groups. Another established difference is also related to the possibility to return to work in different types of occupations.

Rather few studies have reported on long-term consequences of being involved in an accident as a pedestrian. The long-term consequences can be investigated from several different perspectives, the present study investigated the long-term consequences in terms of SA and DP. A study from Sweden reported the long-term consequences, in terms of risk of Permanent Medical Impairment (PMI), showing that 25% of the pedestrian falls and 20% of the pedestrian collisions led to a permanent reduction with a PMI of above 1% [21].

The long-term consequences of pedestrian injuries in terms of SA and DP to the best of our knowledge has not been previously studied. There are only a few studies investigating SA and DP in relation to road traffic accidents [27–30]. Sickness absence and DP have so far only been studied for pedestrians in our previous study, a cross sectional study from Sweden, examining the risk of new SA in connection to the accident, but with no follow-up or information on the duration of SA [8]. That study found that 20% had a new SA in connection to the accident and that 18% had ongoing SA or full-time DP at the time of the accident [8]. Even though those that were on DP throughout follow-up were excluded in the present study, the eight clusters of SA and DP could be comparable to our cross-sectional study [8]. Furthermore, the present study provides a much clearer picture of the SA and DP development during the years after the accident. Separating SA diagnoses into injury diagnoses and other diagnoses also helps in understanding what SA is due to the accident and what could be due to other circumstances. The distinction between the cluster “Episodic SA” and “Both SA due to injury diagnoses and other diagnoses” could not have been made without the possibility to identify SA with injury diagnoses. The pedestrians in the cluster “Episodic SA” had a SA spell due to injury diagnosis in connection to the accident and then one more SA spell due to an injury diagnosis, while the pedestrians in the cluster “Both SA due to injury diagnoses and other diagnoses” had first SA due to an injury diagnoses at the time of the accident and then had later SA due to other diagnoses. The SA spell that occurred later during follow-up does not necessarily need to be connected to the accident, especially for the latter cluster. It could be in relation to late effects of the accident (e.g. injuries, musculoskeletal disease, thrombosis, pneumonia or PTSD) but also a new accident (leading to injury) or even another health issue not related to the accident. In relation to other road user groups, a previous study on bicyclists investigated SA and DP after a bicycle crash but did not make distinctions in SA diagnoses [15]. In addition, other studies investigating SA after a road traffic accident did not separate the analyses for different road user groups or did not differentiate different SA diagnoses in the analysis [27–30]. To elucidate the long-term consequences of road traffic accidents in terms of SA and DP, future studies are needed, especially those with a comparison to the general population.

The relationship between occupational factors and SA after a pedestrian accident has also not been investigated in detail. However, an Australian study found that individuals working as plant and machine operators and drivers had a longer duration of work disability after a road traffic accident [28]. In our study, working in Health & social care was associated with all clusters of SA sequences compared to the cluster “No SA”. Accordingly suggesting an association between this occupational sector and SA following an injury as a pedestrian. In addition, working in Construction was associated with the clusters “Immediate SA” and “Episodic SA” and working in Education was associated with the clusters “Both SA due to injury and other diagnoses” and “Other diagnoses long-term”. This may be explained by the difference in workload and various demands the different occupational sectors have. Individuals in some occupations are more likely to have reduced work capacity in relation to an injury, e.g. due to work requirements of being physical active and/or requiring mobility to complete work tasks. Accordingly, sustaining a fracture to the lower extremities in an occupation where you are required to walk and stand up more often lead to SA. It would be beneficial to pay attention to the physical workload of different jobs in interventions to reduce SA and DP following a pedestrian injury. To elucidate this, further studies are also needed to investigate different jobs, physical workload, and other work demands in more detail.

In the present study, the proportion of missing information on occupation was higher in the three clusters “No SA”, “Other diagnoses Long-term SA”, and “DP”. This could be due to that the individuals in the cluster “No SA” to a lower extent have a job and hence are not eligible to SA benefits (e.g. no income from work, unemployment and parental leave) the individuals in this cluster are also slightly younger than in the other clusters (e.g. still studying or not yet having begun their first job). The high proportion of missing in the other two clusters could be owing to the high proportion of these individuals were already on long-term SA and DP the year before the accident, when the information on occupation was assessed. The proportion of individuals in each occupational sector in this study corresponds well to those of the general population in Sweden[31], with the exception of women working in Health & social care where a higher proportion were observed among the injured pedestrians.

### Strengths and limitations

One of the main strengths of this study is the use of high-quality nationwide register data, with total population coverage, several years of follow-up, and that the results were not hampered by recall bias [32]. Another strength is that all pedestrian injuries are included, not only those involving a motor vehicle. Several recent studies stress that pedestrian injuries should be included in the traditional definition of traffic accidents [1, 21, 33]. The present study further strengthens this argument as 71% of the pedestrian accident were due to fall with no other road user involved. The large number of included pedestrians allowed for more detailed analyses investigating important factors for the subsequent patterns of SA.

One limitation is that no data from primary healthcare (to also capture the impact of minor injuries) was included, on the other hand, all injuries from traffic accidents severe enough to require in- or outpatient healthcare were included. which are those more likely to require SA. Another limitation is that only information from one injury diagnosis per pedestrian is taken into consideration, however, the majority had only one such injury (82%). Another limitation is that only individuals that survived the entire follow-up period were included in the study and that those with DP during the entire study period were excluded. These requirements might potentially lead to an underestimation of the total number of injuries and the shorter-term consequences, there were however few that died or emigrated during follow-up (during the three years after the accident). The focus here was the consequences in terms of SA and DP and as such the included individuals should be at risk of receiving SA or DP.

## Conclusions

This nationwide register study of the 11,432 working-aged pedestrians that received in- or specialized outpatient healthcare due to a traffic-related accident observed diverse patterns of SA due to injury diagnoses and SA due to other diagnoses following the accident. Almost half of the pedestrians had no SA during the entire follow up. Compared to this cluster of SA sequences, all other clusters were associated with older age, no university education, having been hospitalized, and working in health and social care. The three clusters of sequences characterised by SA due to an injury diagnosis in direct connection to the accident were associated with women, fractures, lower and upper extremities. A rather divergent population with more older women and more younger men was observed. The distribution of type of injuries as well as occupational sectors were also distinctly different for women and men. These observed differences can be utilized to improve understanding of long-term consequences and prevention of road-traffic accidents among pedestrians.

## Supplementary Information

**Additional file 1. Table A.1.** Measures of cluster partition quality for four to twelve identified clusters of sequences of SA among pedestrians injured in aroad traffic accident, the measures for the chosen number of clusters included in the present study are marked in bold. **Table A.2. **Distributions of the different sociodemographic, occupation, and injury factors in the eight identified clusters of sequences of sickness absence (SA) status/week over 1 year before and 3 years after the date of a pedestrian accident (W_−52_ to W_+156_) among 11,432 individuals aged 20–59 years injured in a traffic-related accident in 2014-2016. **Table A.3.** Crude and mutually adjusted odds ratios (ORs) and 95% confidence intervals (CIs) for different sociodemographic, occupation, and injury factors in each of the eight identified clusters of sequences of sickness absence (SA) status/week over 1 year before and 3 years after the date of a pedestrian accident (W_−52_to W_+156_) among 11,432 individuals aged 20–59 years injured in a traffic-related accident in 2014-2016, using the cluster “No SA” as the reference.  

## Data Availability

The data cannot be made publicly available, according to privacy regulations. According to the General Data Protection Regulation, the Swedish law SFS 2018:218, the Swedish Data Protection Act, the Swedish Ethical Review Act, and the Public Access to Information and Secrecy Act, data can only be made available, after legal review, for researchers who meet the criteria for access to this type of sensitive and confidential data. Readers may contact professor Kristina Alexanderson (kristina.alexanderson@ki.se) regarding the data.

## Abbreviations

DP: Disability pension
SA: Sickness absence
OR: Odds ratio
CI: Confidence interval
