# Supplementary material for: Diagnosis-specific sickness absence among injured working-aged pedestrians: a sequence analysis
Source: BMC Public Health. 2023 Feb 20;23:367. doi: 10.1186/s12889-023-15259-w (PMC9942404; doi:10.1186/s12889-023-15259-w)
Supplement: Supplementary file 1 — Additional file 1. Table A.1. Measures of cluster partition quality for four to twelve identified clusters of sequences of SA among pedestrians injured in aroad traffic accident, the measures for the chosen number of clusters included in the present study are marked in bold. Table A.2. Distributions of the different sociodemographic, occupation, and injury factors in the eight identified clusters of sequences of sickness absence (SA) status/week over 1 year before and 3 years after the date of a pedestrian accident (W−52 to W+156) among 11,432 individuals aged 20–59 years injured in a traffic-related accident in 2014-2016. Table A.3. Crude and mutually adjusted odds ratios (ORs) and 95% confidence intervals (CIs) for different sociodemographic, occupation, and injury factors in each of the eight identified clusters of sequences of sickness absence (SA) status/week over 1 year before and 3 years after the date of a pedestrian accident (W−52to W+156) among 11,432 individuals aged 20–59 years injured in a traffic-related accident in 2014-2016, using the cluster “No SA” as the reference. [file 12889_2023_15259_MOESM1_ESM.docx]

**Appendix**

Table A.1. Measures of cluster partition quality for four to twelve identified clusters of sequences of SA among pedestrians injured in a road traffic accident, the measures for the chosen number of clusters included in the present study are marked in bold.

|  | PBC^1^ | HG^2^ | HGSD^3^ | ASW^4^ | ASWw^5^ | R2^6^ | R2sq^7^ | HC^8^ | CH^9^ | CHsq^10^ |
| --- | --- | --- | --- | --- | --- | --- | --- | --- | --- | --- |
| 4 clusters | 0.63 | 0.77 | 0.77 | 0.58 | 0.58 | 0.50 | 0.60 | 0.09 | 3847.67 | 5781.73 |
| 5 clusters | 0.65 | 0.80 | 0.80 | 0.58 | 0.58 | 0.53 | 0.65 | 0.08 | 3231.57 | 5208.65 |
| 6 clusters | 0.68 | 0.88 | 0.88 | 0.59 | 0.59 | 0.58 | 0.68 | 0.05 | 3199.42 | 4912.38 |
| 7 clusters | 0.70 | 0.90 | 0.90 | 0.60 | 0.60 | 0.60 | 0.71 | 0.04 | 2863.69 | 4627.30 |
| **8 clusters** | **0.71** | **0.92** | **0.92** | **0.61** | **0.61** | **0.62** | **0.73** | **0.04** | **2654.58** | **4500.16** |
| 9 clusters | 0.72 | 0.95 | 0.95 | 0.62 | 0.62 | 0.64 | 0.76 | 0.03 | 2591.15 | 4487.94 |
| 10 clusters | 0.72 | 0.97 | 0.97 | 0.63 | 0.63 | 0.66 | 0.78 | 0.02 | 2467.29 | 4461.19 |
| 11 clusters | 0.72 | 0.97 | 0.97 | 0.64 | 0.64 | 0.67 | 0.79 | 0.02 | 2307.67 | 4295.10 |
| 12 clusters | 0.72 | 0.97 | 0.97 | 0.63 | 0.63 | 0.67 | 0.80 | 0.02 | 2147.82 | 4091.58 |

1 Point Biserial Correlation

2 Hubert’s Gamma
3 Hubert’s Somers’ D

4 Average Silhouette Width
5 Average Silhouette Width (weighted)
6 Pseudo R^2^

7 Pseudo R^2^ squared

8 Hubert’s C
9 Calinski-Harabasz index

10 Calinski-Harabasz index squared

Table A.2. Distributions of the different sociodemographic, occupation, and injury factors in the eight identified clusters of sequences of sickness absence (SA) status/week over 1 year before and 3 years after the date of a pedestrian accident (W_−52_ to W_+156_) among 11432 individuals aged 20–59 years injured in a traffic-related accident in 2014-2016.

|  | **1. No SA** | **2. Immediate SA** | **3. Episodic SA** | **4. Long-term or later SA** | **5. Both SA due to injury and other diagnoses** | **6. Other diagnoses short-term SA** | **7. Other diagnoses long-term SA** | **8. Disability pension** |
| --- | --- | --- | --- | --- | --- | --- | --- | --- |
|  | n (%) | n (%) | n (%) | n (%) | n (%) | n (%) | n (%) | n (%) |
| **All** | 5339 (46.7) | 2046 (17.9) | 445 (3.89) | 369 (3.23) | 796 (6.96) | 1901 (16.63) | 233 (2.04) | 303 (2.65) |
| **Sex** |  |  |  |  |  |  |  |  |
| Women | 2529 (47.37) | 1142 (55.82) | 258 (57.98) | 177 (47.97) | 538 (67.59) | 1244 (65.44) | 166 (71.24) | 158 (52.15) |
| Men | 2810 (52.63) | 904 (44.18) | 187 (42.02) | 192 (52.03) | 258 (32.41) | 657 (34.56) | 67 (28.76) | 145 (47.85) |
| **Age group, years** |  |  |  |  |  |  |  |  |
| 20-24 | 1067 (19.99) | 235 (11.49) | 38 (8.54) | 51 (13.82) | 61 (7.66) | 221 (11.63) | -^1^ | 61 (20.13) |
| 25-34 | 1184 (22.18) | 350 (17.11) | 84 (18.88) | 66 (17.89) | 103 (12.94) | 426 (22.41) | 37 (15.88) | 56 (18.48) |
| 35-44 | 1103 (20.66) | 360 (17.60) | 62 (13.93) | 64 (17.34) | 161 (20.23) | 396 (20.83) | 64 (27.47) | 33 (10.89) |
| 45-54 | 1290 (24.16) | 630 (30.79) | 149 (33.48) | 114 (30.89) | 282 (35.43) | 520 (27.35) | 79 (33.91) | 80 (26.40) |
| 55-59 | 695 (13.02) | 471 (23.02) | 112 (25.17) | 74 (20.05) | 189 (23.74) | 338 (17.78) | 50 (21.46) | 73 (24.09) |
| **Level of education** |  |  |  |  |  |  |  |  |
| Elementary school | 901 (16.88) | 230 (11.24) | 68 (15.28) | 52 (14.09) | 115 (14.45) | 266 (13.99) | 47 (20.17) | 99 (32.67) |
| High school | 2476 (46.38) | 1123 (54.89) | 248 (55.73) | 229 (62.06) | 434 (54.52) | 1016 (53.45) | 122 (52.36) | 148 (48.84) |
| University/College | 1962 (36.75) | 693 (33.87) | 129 (28.99) | 88 (23.85) | 247 (31.03) | 619 (32.56) | 64 (27.47) | 56 (18.48) |
| **Country of birth** |  |  |  |  |  |  |  |  |
| Sweden | 4164 (77.99) | 1707 (83.43) | 360 (80.90) | 303 (82.11) | 656 (82.41) | 1575 (82.85) | 195 (83.69) | 240 (79.21) |
| Not Sweden | 1175 (22.01) | 339 (16.57) | 85 (19.10) | 66 (17.89) | 140 (17.59) | 326 (17.15) | 38 (16.31) | 63 (20.79) |
| **Type of living area** |  |  |  |  |  |  |  |  |
| Cities | 2435 (45.61) | 765 (37.39) | 163 (36.63) | 146 (39.57) | 270 (33.92) | 759 (39.93) | 109 (46.78) | 105 (34.65) |
| Towns and suburbs | 2096 (39.26) | 901 (44.04) | 198 (44.49) | 160 (43.36) | 357 (44.85) | 791 (41.61) | 80 (34.33) | 140 (46.20) |
| Rural areas | 808 (15.13) | 380 (18.57) | 84 (18.88) | 63 (17.07) | 169 (21.23) | 351 (18.46) | 44 (18.88) | 58 (19.14) |
| **Married** |  |  |  |  |  |  |  |  |
| Yes | 1760 (32.96) | 781 (38.17) | 172 (38.65) | 140 (37.94) | 317 (39.82) | 645 (33.93) | 77 (33.05) | 74 (24.42) |
| No | 3579 (67.04) | 1265 (61.83) | 273 (61.35) | 229 (62.06) | 479 (60.18) | 1256 (66.07) | 156 (66.95) | 229 (75.58) |
| **Type of accident** |  |  |  |  |  |  |  |  |
| Collision with pedestrian/bicyclist | 307 (5.75) | 89 (4.35) | 21 (4.72) | 28 (7.59) | 44 (5.53) | 79 (4.16) | 10 (4.29) | 13 (4.29) |
| Collision with motor vehicle | 777 (14.55) | 286 (13.98) | 57 (12.81) | 59 (15.99) | 79 (9.92) | 256 (13.47) | 33 (14.16) | 48 (15.84) |
| Unspecified | 587 (10.99) | 163 (7.97) | 42 (9.44) | 35 (9.49) | 61 (7.66) | 220 (11.57) | 25 (10.73) | 33 (10.89) |
| Fall-snow and ice | 1005 (18.82) | 600 (29.33) | 122 (27.42) | 78 (21.14) | 249 (31.28) | 370 (19.46) | 47 (20.17) | 58 (19.14) |
| Fall-slipping, tripping, and stumbling | 1938 (36.30) | 670 (32.75) | 150 (33.71) | 130 (35.23) | 279 (35.05) | 753 (39.61) | 102 (43.78) | 114 (37.62) |
| Fall-other | 725 (13.58) | 238 (11.63) | 53 (11.91) | 39 (10.57) | 84 (10.55) | 223 (11.73) | 16 (6.87) | 37 (12.21) |
| **Inpatient healhcare** |  |  |  |  |  |  |  |  |
| No | 4921 (92.17) | 1565 (76.49) | 350 (78.65) | 313 (84.82) | 638 (80.15) | 1713 (90.11) | 188 (80.69) | 235 (77.56) |
| Yes | 418 (7.83) | 481 (23.51) | 95 (21.35) | 56 (15.18) | 158 (19.85) | 188 (9.89) | 45 (19.31) | 68 (22.44) |
| **Type of injury** |  |  |  |  |  |  |  |  |
| Fracture | 1549 (29.01) | 1477 (72.19) | 290 (65.17) | 109 (29.54) | 503 (63.19) | 464 (24.41) | 86 (36.91) | 109 (35.97) |
| Dislocation | 177 (3.32) | 73 (3.57) | 16 (3.60) | 13 (3.52) | 29 (3.64) | 48 (2.52) | -^1^ | -^1^ |
| Sprains and strains | 890 (16.67) | 182 (8.90) | 57 (12.81) | 70 (18.97) | 119 (14.95) | 353 (18.57) | 23 (9.87) | 30 (9.90) |
| Internal | 392 (7.34) | 82 (4.01) | 17 (3.82) | 33 (8.94) | 34 (4.27) | 172 (9.05) | 20 (8.58) | 44 (14.52) |
| External | 2213 (41.45) | 220 (10.75) | 58 (13.03) | 136 (36.86) | 103 (12.94) | 820 (43.14) | 88 (37.77) | 111 (36.63) |
| Other and unspecified | 118 (2.21) | 12 (0.59) | -^1^ | -^1^ | -^1^ | 44 (2.31) | 9 (3.86) | -^1^ |
| **Injured body region** |  |  |  |  |  |  |  |  |
| Head, face and neck | 1394 (26.11) | 155 (7.58) | 32 (7.19) | 83 (22.49) | 61 (7.66) | 474 (24.93) | 49 (21.03) | 97 (32.01) |
| Vertebral column and spinal cord | 89 (1.67) | 35 (1.71) | 10 (2.25) | 15 (4.07) | 13 (1.63) | 41 (2.16) | -^1^ | -^1^ |
| Torso | 312 (5.84) | 68 (3.32) | 18 (4.04) | 22 (5.96) | 31 (3.89) | 147 (7.73) | 15 (6.44) | 17 (5.61) |
| Upper extremities | 1654 (30.98) | 938 (45.85) | 206 (46.29) | 129 (34.96) | 344 (43.22) | 557 (29.30) | 84 (36.05) | 93 (30.69) |
| Lower extremities | 1856 (34.76) | 847 (41.40) | 179 (40.22) | 119 (32.25) | 347 (43.59) | 674 (35.46) | 76 (32.62) | 87 (28.71) |
| Other and unspecified | 34 (0.64) | -^1^ | -^1^ | -^1^ | -^1^ | -^1^ | -^1^ | -^1^ |
| **Season** |  |  |  |  |  |  |  |  |
| Winter | 1820 (34.09) | 835 (40.81) | 165 (37.08) | 126 (34.15) | 331 (41.58) | 653 (34.35) | 85 (36.48) | 118 (38.94) |
| Spring | 1212 (22.70) | 344 (16.81) | 98 (22.02) | 88 (23.85) | 153 (19.22) | 419 (22.04) | 36 (15.45) | 55 (18.15) |
| Summer | 1101 (20.62) | 442 (21.60) | 89 (20.00) | 63 (17.07) | 142 (17.84) | 378 (19.88) | 54 (23.18) | 60 (19.80) |
| Autumn | 1206 (22.59) | 425 (20.77) | 93 (20.90) | 92 (24.93) | 170 (21.36) | 451 (23.72) | 58 (24.89) | 70 (23.10) |
| **Year of accident** |  |  |  |  |  |  |  |  |
| 2014 | 1631 (30.55) | 579 (28.30) | 122 (27.42) | 111 (30.08) | 210 (26.38) | 577 (30.35) | 65 (27.90) | 98 (32.34) |
| 2015 | 1832 (34.31) | 726 (35.48) | 148 (33.26) | 141 (38.21) | 286 (35.93) | 635 (33.40) | 77 (33.05) | 111 (36.63) |
| 2016 | 1876 (35.14) | 741 (36.22) | 175 (39.33) | 117 (31.71) | 300 (37.69) | 689 (36.24) | 91 (39.06) | 94 (31.02) |
| **Occupational sector** |  |  |  |  |  |  |  |  |
| Manufacturing, agriculture, forestry & fishing | 556 (10.41) | 230 (11.24) | 48 (10.79) | 49 (13.28) | 77 (9.67) | 165 (8.68) | -^1^ | 12 (3.96) |
| Construction | 240 (4.50) | 147 (7.18) | 40 (8.99) | 29 (7.86) | 38 (4.77) | 79 (4.16) | -^1^ | -^1^ |
| Trade, transport, hotels & restaurants | 993 (18.60) | 427 (20.87) | 85 (19.10) | 63 (17.07) | 150 (18.84) | 386 (20.31) | 34 (14.59) | 22 (7.26) |
| Finance, communication & cultural service | 1447 (27.10) | 520 (25.42) | 95 (21.35) | 85 (23.04) | 178 (22.36) | 475 (24.99) | 45 (19.31) | 52 (17.16) |
| Education | 409 (7.66) | 210 (10.26) | 44 (9.89) | 33 (8.94) | 96 (12.06) | 215 (11.31) | 21 (9.01) | -^1^ |
| Health & social care | 656 (12.29) | 456 (22.29) | 124 (27.87) | 75 (20.33) | 234 (29.40) | 443 (23.30) | 46 (19.74) | 42 (13.86) |
| Not in work/Unknown | 1038 (19.44) | 56 (2.74) | 9 (2.02) | 35 (9.49) | 23 (2.89) | 138 (7.26) | 75 (32.19) | 161 (53.14) |
| **Private/Public** |  |  |  |  |  |  |  |  |
| Private sector | 2693 (50.44) | 1272 (62.17) | 256 (57.53) | 218 (59.08) | 447 (56.16) | 1073 (56.44) | 92 (39.48) | 67 (22.11) |
| Public sector | 1047 (19.61) | 658 (32.16) | 167 (37.53) | 99 (26.83) | 304 (38.19) | 574 (30.19) | 61 (26.18) | 36 (11.88) |
| Not in work/Unknown | 1599 (29.95) | 116 (5.67) | 22 (4.94) | 52 (14.09) | 45 (5.65) | 254 (13.36) | 80 (34.33) | 200 (66.01) |
| **Type of occupation** |  |  |  |  |  |  |  |  |
| White collar | 1974 (36.97) | 919 (44.92) | 181 (40.67) | 133 (36.04) | 377 (47.36) | 828 (43.56) | 72 (30.90) | 55 (18.15) |
| Blue collar | 1314 (24.61) | 733 (35.83) | 175 (39.33) | 141 (38.21) | 277 (34.80) | 555 (29.20) | 51 (21.89) | 49 (16.17) |
| Not in work/Unknown | 2051 (38.42) | 394 (19.26) | 89 (20.00) | 95 (25.75) | 142 (17.84) | 518 (27.25) | 110 (47.21) | 199 (65.68) |

^1^ Too few: ≤ 8 individuals

Table A.3. Crude and mutually adjusted odds ratios (ORs) and 95% confidence intervals (CIs) for different sociodemographic, occupation, and injury factors in each of the eight identified clusters of sequences of sickness absence (SA) status/week over 1 year before and 3 years after the date of a pedestrian accident (W_−52_ to W_+156_) among 11432 individuals aged 20–59 years injured in a traffic-related accident in 2014-2016, using the cluster “No SA” as the reference.

|  | **2. Immediate SA** | | **3. Episodic SA** | | **4. Long-term or later SA** | | **5. Both SA due to injury and other diagnoses** | | **6. Other diagnoses short-term SA** | | **7. Other diagnoses long-term SA** | | **8. Disability pension** | |
| --- | --- | --- | --- | --- | --- | --- | --- | --- | --- | --- | --- | --- | --- | --- |
|  | Crude OR (95% CI) | Adj OR (95% CI) | Crude OR (95% CI) | Adj OR (95% CI) | Crude OR (95% CI) | Adj OR (95% CI) | Crude OR (95% CI) | Adj OR (95% CI) | Crude OR (95% CI) | Adj OR (95% CI) | Crude OR (95% CI) | Adj OR (95% CI) | Crude OR (95% CI) | Adj OR (95% CI) |
| **Sex** |  |  |  |  |  |  |  |  |  |  |  |  |  |  |
| Women | 1.40 (1.27 - 1.56) | 1.24 (1.08 - 1.42) | 1.53 (1.26 - 1.86) | 1.34 (1.05 - 1.72) | 1.02 (0.83 - 1.27) | 1.01 (0.79 - 1.30) | 2.32 (1.98 - 2.71) | 1.92 (1.58 - 2.33) | 2.10 (1.89 - 2.35) | 2.04 (1.80 - 2.32) | 2.75 (2.06 - 3.68) | 2.62 (1.89 - 3.63) | 1.21 (0.96 - 1.53) | 1.25 (0.96 - 1.63) |
| Men | ref. | ref. | ref. | ref. | ref. | ref. | ref. | ref. | ref. | ref. | ref. | ref. | ref. | ref. |
| **Age group, years** |  |  |  |  |  |  |  |  |  |  |  |  |  |  |
| 20-24 | 0.75 (0.62 - 0.90) | 0.74 (0.60 - 0.91) | 0.50 (0.34 - 0.74) | 0.46 (0.31 - 0.70) | 0.86 (0.59 - 1.25) | 0.76 (0.52 - 1.12) | 0.66 (0.47 - 0.91) | 0.63 (0.45 - 0.89) | 0.58 (0.48 - 0.69) | 0.51 (0.42 - 0.62) | -^1^ | -^1^ | 1.21 (0.83 - 1.75) | 0.87 (0.58 - 1.29) |
| 25-34 | ref. | ref. | ref. | ref. | ref. | ref. | ref. | ref. | ref. | ref. | ref. | ref. | ref. | ref. |
| 35-44 | 1.10 (0.93 - 1.31) | 0.94 (0.77 - 1.13) | 0.79 (0.56 - 1.11) | 0.70 (0.49 - 0.99) | 1.04 (0.73 - 1.48) | 1.00 (0.69 - 1.44) | 1.68 (1.29 - 2.18) | 1.43 (1.08 - 1.88) | 1.00 (0.85 - 1.17) | 1.01 (0.86 - 1.20) | 1.86 (1.23 - 2.81) | 2.04 (1.33 - 3.13) | 0.63 (0.41 - 0.98) | 0.71 (0.45 - 1.12) |
| 45-54 | 1.65 (1.42 - 1.92) | 1.21 (1.01 - 1.45) | 1.63 (1.23 - 2.15) | 1.24 (0.91 - 1.68) | 1.59 (1.16 - 2.17) | 1.43 (1.03 - 1.99) | 2.51 (1.98 - 3.19) | 1.76 (1.36 - 2.28) | 1.12 (0.96 - 1.30) | 1.05 (0.89 - 1.24) | 1.96 (1.32 - 2.92) | 1.99 (1.31 - 3.02) | 1.31 (0.92 - 1.86) | 1.48 (1.02 - 2.15) |
| 55-59 | 2.29 (1.94 - 2.71) | 1.52 (1.24 - 1.86) | 2.27 (1.69 - 3.06) | 1.57 (1.12 - 2.20) | 1.91 (1.35 - 2.70) | 1.76 (1.21 - 2.55) | 3.13 (2.42 - 4.04) | 1.94 (1.45 - 2.58) | 1.35 (1.14 - 1.60) | 1.24 (1.03 - 1.50) | 2.30 (1.49 - 3.56) | 2.32 (1.46 - 3.69) | 2.22 (1.55 - 3.18) | 2.61 (1.77 - 3.86) |
| **Level of education** |  |  |  |  |  |  |  |  |  |  |  |  |  |  |
| Elementary school | 0.72 (0.61 - 0.86) | 1.17 (0.95 - 1.45) | 1.15 (0.85 - 1.56) | 1.83 (1.28 - 2.61) | 1.29 (0.91 - 1.83) | 1.59 (1.07 - 2.36) | 1.01 (0.80 - 1.28) | 1.92 (1.44 - 2.54) | 0.94 (0.79 - 1.10) | 1.46 (1.21 - 1.77) | 1.60 (1.09 - 2.35) | 1.66 (1.06 - 2.59) | 3.85 (2.75 - 5.39) | 2.11 (1.44 - 3.10) |
| High school | 1.28 (1.15 - 1.44) | 1.46 (1.27 - 1.69) | 1.52 (1.22 - 1.90) | 1.70 (1.31 - 2.19) | 2.06 (1.60 - 2.66) | 2.06 (1.55 - 2.73) | 1.39 (1.18 - 1.65) | 1.73 (1.42 - 2.10) | 1.30 (1.16 - 1.46) | 1.46 (1.28 - 1.66) | 1.51 (1.11 - 2.06) | 1.64 (1.16 - 2.31) | 2.09 (1.53 - 2.86) | 1.62 (1.15 - 2.28) |
| University/College | ref. | ref. | ref. | ref. | ref. | ref. | ref. | ref. | ref. | ref. | ref. | ref. | ref. | ref. |
| **Country of birth** |  |  |  |  |  |  |  |  |  |  |  |  |  |  |
| Sweden | ref. | ref. | ref. | ref. | ref. | ref. | ref. | ref. | ref. | ref. | ref. | ref. | ref. | ref. |
| Not Sweden | 0.70 (0.62 - 0.80) | 1.10 (0.94 - 1.29) | 0.84 (0.66 - 1.07) | 1.27 (0.96 - 1.66) | 0.77 (0.59 - 1.02) | 0.89 (0.66 - 1.20) | 0.76 (0.62 - 0.92) | 1.11 (0.89 - 1.38) | 0.73 (0.64 - 0.84) | 0.85 (0.73 - 0.99) | 0.69 (0.49 - 0.98) | 0.47 (0.32 - 0.69) | 0.93 (0.70 - 1.24) | 0.56 (0.40 - 0.78) |
| **Type of living area** |  |  |  |  |  |  |  |  |  |  |  |  |  |  |
| Cities | ref. | ref. | ref. | ref. | ref. | ref. | ref. | ref. | ref. | ref. | ref. | ref. | ref. | ref. |
| Towns and suburbs | 1.37 (1.22 - 1.53) | 1.17 (1.03 - 1.34) | 1.41 (1.14 - 1.75) | 1.17 (0.93 - 1.47) | 1.27 (1.01 - 1.60) | 1.08 (0.85 - 1.38) | 1.54 (1.30 - 1.82) | 1.27 (1.06 - 1.53) | 1.21 (1.08 - 1.36) | 1.14 (1.01 - 1.29) | 0.85 (0.64 - 1.14) | 0.77 (0.57 - 1.05) | 1.55 (1.20 - 2.01) | 1.51 (1.15 - 1.99) |
| Rural areas | 1.50 (1.29 - 1.73) | 1.24 (1.04 - 1.47) | 1.55 (1.18 - 2.04) | 1.19 (0.88 - 1.60) | 1.30 (0.96 - 1.77) | 1.00 (0.73 - 1.38) | 1.89 (1.53 - 2.32) | 1.51 (1.20 - 1.90) | 1.39 (1.20 - 1.62) | 1.29 (1.10 - 1.51) | 1.22 (0.85 - 1.74) | 1.04 (0.71 - 1.52) | 1.67 (1.20 - 2.32) | 1.55 (1.09 - 2.19) |
| **Married** |  |  |  |  |  |  |  |  |  |  |  |  |  |  |
| Yes | ref. | ref. | ref. | ref. | ref. | ref. | ref. | ref. | ref. | ref. | ref. | ref. | ref. | ref. |
| No | 0.80 (0.72 - 0.89) | 1.09 (0.96 - 1.25) | 0.78 (0.64 - 0.95) | 1.12 (0.90 - 1.41) | 0.80 (0.65 - 1.00) | 0.93 (0.73 - 1.19) | 0.74 (0.64 - 0.87) | 1.19 (1.00 - 1.41) | 0.96 (0.86 - 1.07) | 1.21 (1.07 - 1.37) | 1.00 (0.75 - 1.32) | 1.34 (0.99 - 1.81) | 1.52 (1.16 - 1.99) | 1.26 (0.93 - 1.72) |
| **Type of accident** |  |  |  |  |  |  |  |  |  |  |  |  |  |  |
| Collision with pedestrian/bicyclist | 0.84 (0.65 - 1.08) | 1.28 (0.96 - 1.70) | 0.88 (0.55 - 1.42) | 1.36 (0.83 - 2.24) | 1.36 (0.89 - 2.08) | 1.60 (1.03 - 2.48) | 1.00 (0.71 - 1.40) | 1.70 (1.18 - 2.45) | 0.66 (0.51 - 0.86) | 0.78 (0.60 - 1.03) | 0.62 (0.32 - 1.20) | 0.86 (0.44 - 1.69) | 0.72 (0.40 - 1.29) | 0.87 (0.47 - 1.60) |
| Collision with motor vehicle | 1.06 (0.91 - 1.25) | 1.54 (1.26 - 1.87) | 0.95 (0.69 - 1.30) | 1.39 (0.98 - 1.98) | 1.13 (0.82 - 1.56) | 1.23 (0.87 - 1.73) | 0.71 (0.54 - 0.92) | 1.12 (0.84 - 1.50) | 0.85 (0.72 - 1.00) | 0.95 (0.79 - 1.14) | 0.81 (0.54 - 1.21) | 0.87 (0.56 - 1.35) | 1.05 (0.74 - 1.49) | 0.91 (0.62 - 1.33) |
| Unspecified | 0.80 (0.66 - 0.98) | 1.06 (0.85 - 1.32) | 0.92 (0.65 - 1.32) | 1.23 (0.85 - 1.79) | 0.89 (0.61 - 1.31) | 0.93 (0.63 - 1.39) | 0.72 (0.54 - 0.97) | 1.03 (0.76 - 1.41) | 0.96 (0.81 - 1.15) | 1.08 (0.90 - 1.30) | 0.81 (0.52 - 1.27) | 0.93 (0.59 - 1.49) | 0.96 (0.64 - 1.42) | 0.93 (0.61 - 1.42) |
| Fall-snow and ice | 1.73 (1.51 - 1.97) | 1.25 (1.05 - 1.48) | 1.57 (1.22 - 2.02) | 1.20 (0.89 - 1.61) | 1.16 (0.87 - 1.55) | 1.08 (0.77 - 1.50) | 1.72 (1.43 - 2.08) | 1.25 (1.00 - 1.57) | 0.95 (0.82 - 1.10) | 0.93 (0.79 - 1.11) | 0.89 (0.62 - 1.27) | 0.79 (0.52 - 1.19) | 0.98 (0.71 - 1.36) | 0.85 (0.58 - 1.24) |
| Fall-slipping, tripping, and stumbling | ref. | ref. | ref. | ref. | ref. | ref. | ref. | ref. | ref. | ref. | ref. | ref. | ref. | ref. |
| Fall-other | 0.95 (0.80 - 1.13) | 1.06 (0.87 - 1.29) | 0.94 (0.68 - 1.31) | 1.12 (0.79 - 1.58) | 0.80 (0.56 - 1.16) | 0.86 (0.59 - 1.25) | 0.80 (0.62 - 1.04) | 1.10 (0.83 - 1.45) | 0.79 (0.67 - 0.94) | 0.97 (0.81 - 1.16) | 0.42 (0.25 - 0.72) | 0.57 (0.33 - 0.99) | 0.87 (0.59 - 1.27) | 0.93 (0.62 - 1.40) |
| **Inpatient healhcare** |  |  |  |  |  |  |  |  |  |  |  |  |  |  |
| No | ref. | ref. | ref. | ref. | ref. | ref. | ref. | ref. | ref. | ref. | ref. | ref. | ref. | ref. |
| Yes | 3.62 (3.14 - 4.17) | 3.13 (2.62 - 3.74) | 3.20 (2.49 - 4.09) | 3.21 (2.40 - 4.29) | 2.11 (1.56 - 2.85) | 2.46 (1.75 - 3.46) | 2.92 (2.39 - 3.56) | 2.86 (2.26 - 3.63) | 1.29 (1.08 - 1.55) | 1.53 (1.25 - 1.88) | 2.82 (2.01 - 3.96) | 2.77 (1.87 - 4.09) | 3.41 (2.55 - 4.54) | 2.47 (1.77 - 3.45) |
| **Type of injury** |  |  |  |  |  |  |  |  |  |  |  |  |  |  |
| Fracture | 9.59 (8.21 - 11.2) | 6.81 (5.71 - 8.13) | 7.14 (5.35 - 9.54) | 4.91 (3.57 - 6.75) | 1.15 (0.88 - 1.49) | 0.91 (0.68 - 1.23) | 6.98 (5.59 - 8.71) | 4.65 (3.63 - 5.96) | 0.81 (0.71 - 0.92) | 0.73 (0.63 - 0.85) | 1.40 (1.03 - 1.89) | 0.94 (0.66 - 1.35) | 1.40 (1.07 - 1.84) | 1.11 (0.80 - 1.54) |
| Dislocation | 4.15 (3.06 - 5.63) | 2.98 (2.13 - 4.15) | 3.45 (1.94 - 6.13) | 2.37 (1.29 - 4.35) | 1.20 (0.66 - 2.15) | 0.94 (0.51 - 1.74) | 3.52 (2.27 - 5.46) | 2.60 (1.63 - 4.16) | 0.73 (0.53 - 1.02) | 0.75 (0.53 - 1.06) | -^1^ | -^1^ | -^1^ | -^1^ |
| Sprains and strains | 2.06 (1.67 - 2.54) | 1.45 (1.15 - 1.83) | 2.44 (1.68 - 3.55) | 1.69 (1.13 - 2.52) | 1.28 (0.95 - 1.73) | 1.12 (0.79 - 1.58) | 2.87 (2.18 - 3.78) | 1.90 (1.41 - 2.57) | 1.07 (0.92 - 1.24) | 0.93 (0.78 - 1.10) | 0.65 (0.41 - 1.04) | 0.62 (0.37 - 1.03) | 0.67 (0.45 - 1.01) | 0.90 (0.56 - 1.43) |
| Internal | 2.10 (1.60 - 2.77) | 3.08 (2.17 - 4.37) | 1.66 (0.95 - 2.87) | 3.04 (1.52 - 6.08) | 1.37 (0.92 - 2.03) | 1.32 (0.82 - 2.11) | 1.86 (1.25 - 2.79) | 3.10 (1.86 - 5.17) | 1.18 (0.97 - 1.44) | 1.27 (1.01 - 1.61) | 1.28 (0.78 - 2.11) | 1.57 (0.86 - 2.87) | 2.24 (1.55 - 3.22) | 1.93 (1.23 - 3.03) |
| External | ref. | ref. | ref. | ref. | ref. | ref. | ref. | ref. | ref. | ref. | ref. | ref. | ref. | ref. |
| Other and unspecified | 1.02 (0.56 - 1.88) | 1.44 (0.76 - 2.75) | -^1^ | -^1^ | -^1^ | -^1^ | -^1^ | -^1^ | 1.01 (0.71 - 1.44) | 1.15 (0.79 - 1.67) | 1.92 (0.94 - 3.90) | 2.38 (1.09 - 5.18) | -^1^ | -^1^ |
| **Injured body region** |  |  |  |  |  |  |  |  |  |  |  |  |  |  |
| Head, face and neck | ref. | ref. | ref. | ref. | ref. | ref. | ref. | ref. | ref. | ref. | ref. | ref. | ref. | ref. |
| Vertebral column and spinal cord | 3.54 (2.31 - 5.41) | 1.53 (0.92 - 2.55) | 4.90 (2.33 - 10.3) | 2.74 (1.16 - 6.46) | 2.83 (1.57 - 5.11) | 3.13 (1.59 - 6.15) | 3.34 (1.77 - 6.30) | 1.79 (0.86 - 3.70) | 1.35 (0.92 - 1.99) | 1.77 (1.16 - 2.72) | -^1^ | -^1^ | -^1^ | -^1^ |
| Torso | 1.96 (1.44 - 2.67) | 1.41 (0.97 - 2.04) | 2.51 (1.39 - 4.54) | 2.11 (1.08 - 4.12) | 1.18 (0.73 - 1.92) | 1.27 (0.75 - 2.13) | 2.27 (1.45 - 3.56) | 1.98 (1.18 - 3.33) | 1.39 (1.11 - 1.73) | 1.64 (1.28 - 2.09) | 1.37 (0.76 - 2.47) | 1.65 (0.87 - 3.15) | 0.78 (0.46 - 1.33) | 0.90 (0.50 - 1.62) |
| Upper extremities | 5.10 (4.24 - 6.13) | 3.26 (2.48 - 4.28) | 5.43 (3.71 - 7.93) | 4.44 (2.60 - 7.58) | 1.31 (0.99 - 1.74) | 1.74 (1.20 - 2.52) | 4.75 (3.59 - 6.30) | 3.27 (2.18 - 4.92) | 0.99 (0.86 - 1.14) | 1.24 (1.03 - 1.49) | 1.44 (1.01 - 2.07) | 2.01 (1.25 - 3.26) | 0.81 (0.60 - 1.08) | 1.21 (0.81 - 1.81) |
| Lower extremities | 4.10 (3.41 - 4.94) | 3.26 (2.49 - 4.28) | 4.20 (2.87 - 6.16) | 4.10 (2.41 - 6.99) | 1.08 (0.81 - 1.44) | 1.32 (0.90 - 1.92) | 4.27 (3.23 - 5.66) | 3.65 (2.44 - 5.48) | 1.07 (0.93 - 1.22) | 1.26 (1.05 - 1.52) | 1.16 (0.81 - 1.68) | 1.64 (1.01 - 2.65) | 0.67 (0.50 - 0.91) | 0.93 (0.62 - 1.41) |
| Other and unspecified | -^1^ | -^1^ | -^1^ | -^1^ | -^1^ | -^1^ | -^1^ | -^1^ | -^1^ | -^1^ | -^1^ | -^1^ | -^1^ | -^1^ |
| **Season** |  |  |  |  |  |  |  |  |  |  |  |  |  |  |
| Winter | 1.14 (1.00 - 1.31) | 0.85 (0.71 - 1.01) | 1.12 (0.86 - 1.47) | 0.85 (0.62 - 1.16) | 1.21 (0.89 - 1.65) | 1.09 (0.77 - 1.54) | 1.41 (1.14 - 1.74) | 0.98 (0.76 - 1.26) | 1.05 (0.90 - 1.21) | 0.96 (0.82 - 1.14) | 0.95 (0.67 - 1.35) | 0.77 (0.51 - 1.14) | 1.19 (0.86 - 1.64) | 1.16 (0.81 - 1.67) |
| Spring | 0.71 (0.60 - 0.83) | 0.65 (0.54 - 0.78) | 1.00 (0.74 - 1.35) | 0.90 (0.66 - 1.23) | 1.27 (0.91 - 1.77) | 1.21 (0.86 - 1.70) | 0.98 (0.77 - 1.25) | 0.85 (0.66 - 1.10) | 1.01 (0.86 - 1.18) | 0.98 (0.83 - 1.16) | 0.61 (0.39 - 0.93) | 0.55 (0.35 - 0.86) | 0.83 (0.57 - 1.21) | 0.81 (0.55 - 1.20) |
| Summer | ref. | ref. | ref. | ref. | ref. | ref. | ref. | ref. | ref. | ref. | ref. | ref. | ref. | ref. |
| Autumn | 0.88 (0.75 - 1.03) | 0.84 (0.70 - 1.00) | 0.95 (0.71 - 1.29) | 0.88 (0.64 - 1.21) | 1.33 (0.96 - 1.86) | 1.29 (0.92 - 1.80) | 1.09 (0.86 - 1.39) | 0.99 (0.77 - 1.28) | 1.09 (0.93 - 1.28) | 1.04 (0.88 - 1.23) | 0.98 (0.67 - 1.43) | 0.80 (0.54 - 1.19) | 1.07 (0.75 - 1.52) | 1.02 (0.70 - 1.48) |
| **Year of accident** |  |  |  |  |  |  |  |  |  |  |  |  |  |  |
| 2014 | ref. | ref. | ref. | ref. | ref. | ref. | ref. | ref. | ref. | ref. | ref. | ref. | ref. | ref. |
| 2015 | 1.12 (0.98 - 1.27) | 1.17 (1.01 - 1.36) | 1.08 (0.84 - 1.39) | 1.12 (0.86 - 1.46) | 1.13 (0.87 - 1.46) | 1.20 (0.91 - 1.58) | 1.21 (1.00 - 1.47) | 1.25 (1.01 - 1.54) | 0.98 (0.86 - 1.12) | 0.97 (0.84 - 1.12) | 1.05 (0.75 - 1.48) | 0.91 (0.62 - 1.32) | 1.01 (0.76 - 1.33) | 0.83 (0.60 - 1.14) |
| 2016 | 1.11 (0.98 - 1.26) | 1.08 (0.93 - 1.26) | 1.25 (0.98 - 1.59) | 1.18 (0.91 - 1.53) | 0.92 (0.70 - 1.20) | 0.94 (0.71 - 1.25) | 1.24 (1.03 - 1.50) | 1.19 (0.97 - 1.46) | 1.04 (0.91 - 1.18) | 1.01 (0.88 - 1.16) | 1.22 (0.88 - 1.68) | 1.00 (0.70 - 1.44) | 0.83 (0.62 - 1.12) | 0.74 (0.54 - 1.02) |
| **Occupational sector** |  |  |  |  |  |  |  |  |  |  |  |  |  |  |
| Manufacturing, agriculture, forestry & fishing | 1.15 (0.96 - 1.38) | 0.97 (0.78 - 1.20) | 1.31 (0.92 - 1.89) | 1.11 (0.74 - 1.65) | 1.50 (1.04 - 2.16) | 1.10 (0.74 - 1.63) | 1.13 (0.85 - 1.50) | 0.96 (0.70 - 1.31) | 0.90 (0.74 - 1.11) | 0.85 (0.68 - 1.06) | -^1^ | -^1^ | 0.60 (0.32 - 1.13) | 0.52 (0.27 - 1.01) |
| Construction | 1.70 (1.36 - 2.14) | 1.62 (1.24 - 2.13) | 2.54 (1.71 - 3.76) | 2.35 (1.51 - 3.65) | 2.06 (1.32 - 3.20) | 1.46 (0.91 - 2.35) | 1.29 (0.88 - 1.87) | 1.38 (0.92 - 2.09) | 1.00 (0.76 - 1.32) | 1.04 (0.77 - 1.39) | -^1^ | -^1^ | -^1^ | -^1^ |
| Trade, transport, hotels & restaurants | 1.20 (1.03 - 1.39) | 1.20 (1.00 - 1.44) | 1.30 (0.96 - 1.77) | 1.25 (0.89 - 1.75) | 1.08 (0.77 - 1.51) | 0.86 (0.60 - 1.24) | 1.23 (0.97 - 1.55) | 1.22 (0.94 - 1.59) | 1.18 (1.01 - 1.39) | 1.12 (0.94 - 1.32) | 1.10 (0.70 - 1.73) | 1.17 (0.72 - 1.91) | 0.62 (0.37 - 1.02) | 0.51 (0.30 - 0.87) |
| Finance, communication & cultural service | ref. | ref. | ref. | ref. | ref. | ref. | ref. | ref. | ref. | ref. | ref. | ref. | ref. | ref. |
| Education | 1.43 (1.18 - 1.74) | 1.19 (0.94 - 1.51) | 1.64 (1.13 - 2.38) | 1.20 (0.79 - 1.83) | 1.37 (0.91 - 2.08) | 1.41 (0.89 - 2.25) | 1.91 (1.45 - 2.50) | 1.48 (1.08 - 2.02) | 1.60 (1.32 - 1.95) | 1.45 (1.16 - 1.80) | 1.65 (0.97 - 2.80) | 1.45 (0.80 - 2.61) | -^1^ | -^1^ |
| Health & social care | 1.93 (1.66 - 2.26) | 1.85 (1.53 - 2.25) | 2.88 (2.17 - 3.82) | 2.45 (1.76 - 3.41) | 1.95 (1.41 - 2.69) | 1.97 (1.36 - 2.86) | 2.90 (2.34 - 3.60) | 2.38 (1.85 - 3.07) | 2.06 (1.75 - 2.41) | 1.73 (1.44 - 2.07) | 2.25 (1.48 - 3.43) | 1.99 (1.24 - 3.22) | 1.78 (1.17 - 2.70) | 1.75 (1.10 - 2.79) |
| Not in work/Unknown | 0.15 (0.11 - 0.20) | 0.45 (0.31 - 0.65) | 0.13 (0.07 - 0.26) | 0.32 (0.14 - 0.72) | 0.57 (0.38 - 0.86) | 0.99 (0.57 - 1.73) | 0.18 (0.12 - 0.28) | 0.48 (0.27 - 0.84) | 0.41 (0.33 - 0.50) | 0.61 (0.47 - 0.81) | 2.32 (1.59 - 3.39) | 2.94 (1.57 - 5.52) | 4.32 (3.13 - 5.96) | 1.63 (1.06 - 2.52) |
| **Private/Public** |  |  |  |  |  |  |  |  |  |  |  |  |  |  |
| Private sector | ref. | ref. | ref. | ref. | ref. | ref. | ref. | ref. | ref. | ref. | ref. | ref. | ref. | ref. |
| Public sector | 1.33 (1.18 - 1.50) | 1.14 (0.96 - 1.36) | 1.68 (1.36 - 2.07) | 1.58 (1.16 - 2.16) | 1.17 (0.91 - 1.50) | 1.03 (0.73 - 1.46) | 1.75 (1.49 - 2.06) | 1.15 (0.91 - 1.45) | 1.38 (1.22 - 1.56) | 0.94 (0.79 - 1.11) | 1.71 (1.22 - 2.38) | 1.03 (0.66 - 1.61) | 1.38 (0.92 - 2.09) | 1.00 (0.60 - 1.65) |
| Not in work/Unknown | 0.15 (0.13 - 0.19) | 0.23 (0.17 - 0.30) | 0.14 (0.09 - 0.22) | 0.23 (0.14 - 0.40) | 0.40 (0.29 - 0.55) | 0.46 (0.29 - 0.74) | 0.17 (0.12 - 0.23) | 0.25 (0.16 - 0.37) | 0.40 (0.34 - 0.46) | 0.50 (0.40 - 0.62) | 1.46 (1.08 - 1.99) | 0.48 (0.27 - 0.87) | 5.03 (3.79 - 6.68) | 2.07 (1.35 - 3.20) |
| **Type of occupation** |  |  |  |  |  |  |  |  |  |  |  |  |  |  |
| White collar | ref. | ref. | ref. | ref. | ref. | ref. | ref. | ref. | ref. | ref. | ref. | ref. | ref. | ref. |
| Blue collar | 1.20 (1.06 - 1.35) | 1.93 (1.63 - 2.27) | 1.45 (1.17 - 1.81) | 2.51 (1.88 - 3.34) | 1.59 (1.24 - 2.04) | 1.86 (1.37 - 2.52) | 1.10 (0.93 - 1.31) | 2.03 (1.62 - 2.53) | 1.01 (0.89 - 1.14) | 1.35 (1.15 - 1.58) | 1.06 (0.74 - 1.53) | 1.99 (1.29 - 3.07) | 1.34 (0.90 - 1.98) | 1.48 (0.94 - 2.33) |
| Not in work/Unknown | 0.41 (0.36 - 0.47) | 1.05 (0.87 - 1.26) | 0.47 (0.36 - 0.61) | 1.42 (1.02 - 1.96) | 0.69 (0.52 - 0.90) | 1.17 (0.83 - 1.66) | 0.36 (0.30 - 0.44) | 1.05 (0.81 - 1.35) | 0.60 (0.53 - 0.68) | 1.23 (1.04 - 1.46) | 1.47 (1.09 - 1.99) | 2.48 (1.62 - 3.78) | 3.48 (2.57 - 4.72) | 2.13 (1.38 - 3.29) |

^1^ Too few: ≤ 8 individuals
